# Case Report: The role of *Panchagavya* and *Panchakarma* treatment in the management of radiotherapy and chemotherapy side effects in infiltrative ductal carcinoma

**DOI:** 10.12688/f1000research.139233.1

**Published:** 2023-10-20

**Authors:** Punam Sawarkar, Gaurav Sawarkar, Nandini Bhojraj

**Affiliations:** 1 Mahatma Gandhi Ayurved College Hospital and Research Centre, Datta Meghe Institute of Higher Education and Research, Wardha, Maharashtra, 442001, India; 2 Consultant, Go Anusandhana Kendra, Deolapar, Maharashtra, 441401, India

**Keywords:** Breast Carcinoma, Chemotherapy, Panchagavya, Panchakarma, Radiotherapy

## Abstract

The incidence rate of infiltrative ductal carcinoma of the breast is increasing worldwide. Chemo- and radiotherapy are commonly used after the surgical intervention for radical cure. The occurrence of various side-effects of these chemo-radiation therapies creates much discomfort to the patient. Therefore, the compliance rate of the patient towards their adoption becomes poor, or the patient is highly affected by their associated side effects in unavoidable circumstances. It is imperative to study the effect of safe, alternative options such as *Panchakarma* and *Panchagavya* treatment, which are widely used in clinical practice as an adjuvant therapy in various types of carcinoma. This report presents the case of a 31-year-old female patient diagnosed with right infiltrative ductal carcinoma of the breast, and who was advised to undergo chemo-radiation therapy after surgical intervention. However, as soon as she finished the first chemo cycle, she suffered from palpitations, loss of appetite, nausea, vomiting, severe restlessness, and hot flushes. She was rushed to the *Kamdhenu Panchgavya Ayurvedic* Clinic of *Govigyan Anusandhan Kendra.* Specific *Panchagvya Chikitsa,* along with *Mrudu Shodhana* based on *Ayurvedic* principles, was prescribed to her ( *Kamdhenu Gomutra Ark, Laghusutshekhar Ras*, *Panchagavya Ghrita, Panchatikta Kshir Vasti, Anuvasana Vasti with Panchgavya Ghrita*) throughout her total rounds of chemo- and radiotherapy. After starting the above-said *Ayurvedic* treatment, the patient experienced significant relief in all symptoms. Her full six sittings of chemo- and radiotherapy were smoothly completed without causing any untoward effect. The selected combination of *Ayurveda* medicines gave relief in all symptoms induced by chemo- and radiotherapy therapy due to their *Vatanulomak, Pittghna, Dahahara, Balya* and *Rasayana* properties. The present case study shows that the *Panchakarma* and *Panchagavya* treatment is effective to subside the side effects induced by chemo- and radiotherapy, and improve the compliance rate of the patients towards these conventional therapies.

## Introduction

Infiltrating ductal carcinoma (IDC), also called invasive ductal carcinoma, is a carcinoma involving the milk duct. It is the most typical breast carcinoma (80 % of total incidences of breast carcinoma). ^1^ Its spread takes place through the lymph nodes to other body tissues. ^2^ The number of patients of this type of carcinoma of breast are increasing day by day, both globally and in India. ^3^ Though the carcinoma of breast is considered as better compared to other cancers from a treatment and prognosis perspective, the recurrence of especially infiltrative ductal carcinoma is a common entity. ^4^ Therefore, its radical cure treatment in the form of chemo- and radiotherapy is highly appreciated, preceded by surgical intervention. However, there are multiple side-effects of these chemo-radiation therapies such as body aches, nausea, vomiting, and a severe burning sensation, which is quite troublesome for the patient as these symptoms adversely affect the quality of life of the patient. ^5^ As a result, many patients decline to undergo further courses and may be deprived of their total expected therapeutic outcome. ^6^ In these circumstances, the pathogenesis of the primary disease may worsen or may even lead to the death of the patient due to further metastatic changes.

Moreover, modern science is limited in preventing such complications; it is difficult to target cancer stem cells (CSCs), drug resistance properties of cancer stem cells make them immune to anticancer drugs, a lack of cancer epigenetic profiling and specificity of existing epi-drugs, problems associated with cancer diagnosis make it difficult to treat, a lack of effective biomarkers for cancer diagnosis and prognosis, and there are limitations of conventional chemotherapeutic agents. ^7^ Therefore, it is the need of the hour to search for some safe alternative options in *Ayurveda* to manage these complications effectively. *Panchagavya* treatment and *Mrudu Shodhana,* sourced from *Ayurveda* medicine, seem to be a ray of hope for such patients which is demonstrated through this case report.

## Case report

### Patient information

The 31-year-old married female patient, housewife, with a diagnosis of right infiltrative ductal carcinoma of the breast, who had a mastectomy three weeks before her first chemotherapy session, approached *Go-Anusandhana Chikitsasalaya*, Nagpur, in February 2014. According to her history, she was experiencing issues such as palpitations, loss of appetite, nausea, and vomiting. These symptoms began after the first chemo session, and after the third session she additionally experienced hot flushes, constipation, burning micturition, and sensations of burning in the anal region. Thus, she was unable to consume any type of food. A detailed history of the patient is presented in Table 1. Upon examination at her first visit to the outpatient department, the general condition of the patient was fair, no icterus/swelling was found, mild pallor, palpable axillary lymphadenopathy, and blood pressure was 90/70 mm of Hg. *Prakriti* of patient was *Vatapradhan Pittaj Prakriti. Ashatavidha Parikshana* results are mentioned in Table 2.

**Table 1.** Patient past-present-drug and family history.

**Past medical history**	Jaundice before six years No other major medical/surgical illness
**Personal history**	Diet: vegetarian Sleep: disturbed Occupation: housewife **Menstrual history:** Menarche at the age of 13 years Regular (30 days cycle) for four days, painless with moderate bleeding **Obstetric history:** G1P1A0 (normal delivery but a mentally disabled male child)
**Drug History**	Chemotherapy
**Family history**	Father: no specific history Mother: K/C/O HTN Siblings: normal Paternal uncle: K/C/O carcinoma of stomach

**Table 2.** Ashatavidha Parikshana.

S.N.	Head	Observation	S.N.	Head	Observation
**1**	*Nadi* (pulse)	64/min ( *Vata-Pittaja*)	**5**	*Shabda* (speech)	*Spashta* (clear)
**2**	*Mala* (stool)	Constipated (after third chemo)	**6**	*Sparsha* (touch)	*Ushna*
**3**	*Mutra* (urine)	Burning micturition	**7**	*Druka* (vision)	Good
**4**	*Jivha* (tongue)	*Sama*	**8**	*Akruti (*posture)	*Madhyam*

She was given Ayurvedic medication as adjuvant therapy for infiltrative ductal cancer and to address these side effects of chemotherapy. The patient self-reported that the intensity of the above symptoms was controlled with the prescribed medicines: *Kamdhenu Gomutra Ark* (distilled cow urine) 10 ml twice a day with the dilution of 100 ml water on an empty stomach; *Laghusutashekhar Rasa* (comprising of *Swarna gairika* – purified red ochre, iron oxide; *Shunthi* – ginger rhizome, Zingiber officinalis; *Nagavalli* – betel leaf juice extract, piper betel) 125 mg tablets twice a day with clarified butter and candy sugar *; Panchagavya Ghrita* 10 ml twice a day with each meal when hungry; *Panchatikta Kshir Vasti* 50 ml before the day of chemotherapy and alternating three enemas sittings after three days of chemotherapy; *Matra Vasti* with *Panchagavya Ghrita* 60 ml once a week after food for six months.

However, eight weeks after completing her third chemo round, she visited the out-patient department again suffering from additional symptoms such as severe hot flushes (all over the body), constipation, burning micturition and burning sensation in the anal region, and her quality of life was also severely affected. Therefore, specific *Panchakarma (Mrudu Shodhana*) of 60 ml *Matra Basti* with *Panchagavya Ghrut* was prescribed, which was continued throughout her chemo- and radiotherapy. After adding this, encouraging results were found in all signs and symptoms, and all sittings of chemo-radiotherapy were completed smoothly without any undue effect.

### Timeline and diagnostic assessment

The patient first reported to the outpatient department with a pre-existing diagnosis of breast infiltrative ductal carcinoma. The oncologist did not determine the patient’s prognosis due to the unknown metastases in the body. Pathological investigations of the patient carried out for the diagnosis are described in Table 3.

**Table 3.** Method of diagnosis and investigation.

S.N.	Method of diagnosis/investigations	Timeline	Impression/remark
1	Self-recognition	16/02/2014	The feeling of a lump in the right breast
2	Chest PA view	17/02/2014	NAD
3	USG (abdomen & pelvis)	17/02/2014	Bulky uterus with small cystic lesions in left ovary
4	Skeletal scintigraphy	18/02/2014	Scan negative for skeletal metastasis
5	Bilateral mammography	17/02/2014	Irregular speculated high-density 3.5*2.5 cm lump in the upper right breast ACR-BI-RADS Category
6	Biopsy (histopathology)	21/02/2014	+ ve for malignancy Muscle bundles with a small focus of tumor showing infiltrating duct carcinoma
7	FNAC (Rt axillary lymph node)	21/02/2014	Scattered polymorphs in hemorrhagic background
8	Surgical intervention	27/02/2014	Mastectomy
9	First chemo	20/03/2014	Side-effects started

After the initial chemo treatments, the patient reported to the outpatient department with palpitation, lack of appetite, nausea, and vomiting. *Kamdhenu Gomutra Ark, Laghusutshekhar,* and *Panchagavya Ghrit* were prescribed for primary treatment. After 4 weeks between chemo rounds, the patient felt better. After 8 weeks, the third chemo session, the patient reported additional symptoms such hot flushes, constipation, burning micturition, and anal burning. *Panchatikta Kshir Vasti* was administered before chemo, three enemas three days after chemo, and *Matra Vasti* with *Panchagavya Ghrita* once a week after eating. After six months, the patient claimed improvement from palpitation, loss of appetite, nausea, vomiting, hot flushes, constipation, burning micturition, and anal burning.

### Therapeutic intervention

In this therapeutic intervention, palliative treatment and purification were given: prescribed medicines: *Kamdhenu Gomutra Ark* (distilled cow urine) 10 ml twice a day with the dilution of 100 ml water on an empty stomach; *Laghusutashekhar Rasa* 3 125 mg tablets twice a day with clarified butter and candy sugar *; Panchagavya Ghrita* (Ghrit prepared from cow milk, curd, clarified butter, urine, and dung) 10 ml twice a day with each meal when hungry; *Panchatikta Kshir Vasti* (enema of cow milk treated with Azadirachta indica (stem bark), Tinospora cordifolia (stem), Adhatoda vasica (root), Solanum xanthocarpum (root) and Trichosanthes dioica (aerial parts)) 50 ml before the day of chemotherapy and alternating three enemas after three days of chemotherapy; *Matra Vasti* with *Panchagavya Ghrita* (medicated clarified butter enema with Panchagavya) 60 ml once a week after food for six months.

Palliative treatment increases the quality of life of patients and their families who are dealing with the hardships of a life-threatening disease, and the purification method helps to detoxify the body for better improvement in quality of life. Details of the types of therapeutic intervention and administration of therapeutic intervention applied in this case are depicted in Table 4.

**Table 4.** Details of therapeutic intervention applied.

S.N.	Medicine	Dose	Frequency	Time of administration	After drink	Duration
1	*Kamdhenu Gomutra Ark* (Distilled cow urine)	10 ml	Twice a day	With empty stomach	With the dilution of 100ml water	For six months
2	*Laghusutashekhar Rasa*	3tabs	Twice a day	10 a.m. - 2.p.m.	*Goghrita+* candy sugar	
3	*Panchagavya Ghrita* (Ghrit prepared from cow milk, curd, clarified butter, urine, and dung)	10ml	Twice a day	At the time of hunger	with each meal	
4	*Panchatikta Kshir Vasti* [Enema of Cow milk treated with Azadirachta indica (stem bark), Tinospora cordifolia (stem), Adhatoda vasica (root), Solanum xanthocarpum (root) and Trichosanthes dioica (aerial parts)]	50 ml *(Godugdha 25ml +Pancha tikta Ghrita 25 ml)*	-	Before the day of chemo & alternate three enemas after three days of chemo	-	Six months
5	*Matra Vasti* with *Panchagavya Ghrita* [Medicated clarified butter enema with Panchagavya]	60 ml	-	Once a week after the food	-	Six months

### Follow-up and therapeutic outcome

After prescribing the above treatment plan compromising of *Mrudu Shodhana (Ksheervasti +Anuvasana Vasti*) with *Panchagavya Chikitsa* for 6 months, the patient felt significant relief in all side effects of the chemo- and radiotherapy and the conventional therapies were completed smoothly and successfully. Moreover, to date, there is no recurrence of the disease or any complication in the form of metastasis. The symptoms like palpitations, lack of appetite, nausea, vomiting, hot flushes, constipation, burning micturition, and burning sensation in the anal region reduced remarkably. Therapeutic outcomes observed in this patient are mentioned in Table 5.

**Table 5.** Therapeutic outcome.

S.N.	Clinical findings	Before Treatment	After one month	After two months	After three months	After four months	After five months	After six months
1	Palpitation	4	2	1	0	0	0	0
2	Loss of appetite	3	1	1	1	0	0	0
3	Nausea	4	1	1	1	0	0	0
4	Vomiting	4	0	0	0	0	0	0
5	Hot flushes (All over body)	-	-	-	4	1	0	0
6	Constipation	-	-	-	4	0	0	0
7	Burning micturition	-	-	-	4	0	0	0
8	Burning sensation in anal region	-	-	-	3	0	0	0

Note: 0- Absent, 1-Mild, 2-Moderate, 3-Severe, 4-Extrem Severe.

## Discussion

*Kamdhenu Gomutra Arka* (distilled cow urine) is one of the most effective medicines among *Panchagavya* formulations. ^8^ Due to its highly concentrated nature as a result of the specific method of preparation and original properties of *Gomutra,* it is highly *Kaphaghn*a and *Agnidipaka* in nature, which increases the patient’s appetite. ^9^ According to the modern perspective, potassium in *Gomutra Arka* is responsible for its effect on appetite. ^9^ *Ruksha Guna* decreases the excess *Drava Guna* of *Pitta* which results in relief of nausea. The sodium in *Gomutra Arka* relieves the hyperacidity. Mainly, *Gomutra* corrects the *Kledak Kapha Dushti,* which is the leading cause of *Chhardi,* i.e., vomiting. Due to its *Kaphaghna* properties, it is useful in *Kapha-*predominant conditions in *Granthi* and *Arbuda.* It is highly effective in various carcinomatous conditions due to the anti-cancer properties of *Gomutra.* ^10^ *Gomutra* is diuretic in nature; it eliminates the excess *Kleda* (toxic materials induced by chemotherapeutic agents) and also decreases the burning sensation during urination. ^11^ It also decreases hematological toxicities induced by chemo and radiation therapies. ^12^^,^^13^

*Gomutra* plays a crucial role in immune modulation by enhancing the activity of macrophages, lymphocytes (both T and B cells), humoral cellular immunity, and cytokines (interleukin 1 and 2), and works as an adjuvant therapy with the conventional modalities. ^14^ It also protects the cell from getting damaged by free radicals, which can cause tumor cell growth or recurrence. ^14^

*Laghusutashekhar Rasa* is the combination of herbs and minerals that consists of *Gairik* responsible for the *Pitta Shamana* effect due to its *Shita Virya*, which subsides the symptoms of *Pitta Prakopaka* such as *Sarvang Daha* (hot flushes), *Mootra Daha* and *Guda Daha.* It absorbs the excess *Drava Guna* of vitiated *Pitta,* which occurs due to *Ushna Guna* of the chemotherapeutic agent. Therefore, it decreases the sensation of nausea and reduces the frequency of vomiting ^15^ and is highly recommended for acid peptic disease and lack of appetite. It also pacifies *Vata* due to its *Madhura Rasa, Snigdha, and Madhur Vipaka.* ^16^ As it contains *Go–Ghrita,* which is *Agnidipaka* in nature, it results in increased appetite. ^17^

*Panchagavya Ghrita* (Ghrit prepared from cow milk, curd, clarified butter, urine, and dung) is the medicated *Ghrita* that pacifies the *Pitta* and *Kapha.* ^18^ In this case, it was used in the forms of *Shamana Snehapana* and *Matra Vasti.* *Shamana Snehapana,* with *Panchagavya Ghrita,* acts as a carrier that can facilitate the drug or its pharmacological properties to get into the cells, enhancing the effects of *Shamana* drugs. ^19^ It processes free radicals as a result of the anti-oxidant effects due to vitamins A, C, and E, and fatty acids present in it. It is especially recommended for liver disorders. ^20^ It acts as the best immuno-modulator through its anti-oxidant properties. ^20^ It also has anti-cancer properties and helps to remove the hematogenous toxins induced by chemotherapeutic agents. ^21^^–^^22^ The neuroprotective action of *Panchagvaya Ghrita* is demonstrated by *Sawarkar G et al.* (2018), resulting in the relief in hot flushes. ^23^

*Matra Vasti* with *Panchgavya Ghrita* (enema of cow milk treated with Azadirachta indica (stem bark), Tinospora cordifolia (stem), Adhatoda vasica (root), Solanum xanthocarpum (root) and Trichosanthes dioica (aerial parts)) acts as a mild type of purification and can be adopted to induce harmony between the vitiated *Dosha* and *Dhatu*, which can normalize the *Vata* without causing any deterioration in the status of the person. ^24^ While *Shodhan Vasti* is contraindicated in cancer treatment due to the complexity of the disease, *Shaman Vasti* can be used with cancer patients as it is a mild type of *Vasti.* Moreover, side effects induced by chemo- and radiotherapy mainly affect the *Rasavaha, Annavaha* and *Pursihvaha Strotas* by causing vitiation of *Vata* and *Pitta* primarily. Therefore, *Panchagavya Ghrita,* which mainly is *Pitta Shamaka*, also brings the *Vatanulomama* (especially *ApanVayu*) due to *Snigdha Guna* of *Ghrita.* As a result, it corrects the *Saman Vayu* and *Pachak Pitta.* It reverts the normalcy of *Annavaha Strotas* by improving appetite. ^25^

*Panchtikta Kshir Vasti* (medicated clarified butter enema with *Panchagavya*) is recommended for *Pitta* predominant and degenerative conditions due to its *Pitta Shamaka, Kledahara, Balya*, and *Rasayan* properties. ^26^ In clinical practice, it has significant results in *Amlapittta Daha, Astha-Majjagata Dushti Vikara, and Dhatukshayajanaya Vata Vyadhi.* ^27^ The *Kaphaghna* and *Sukshma* properties of *Panchtikta (Guduchi, Patol, Kantakari, Nimba and Vasa*) reduces the inflammation in gastric mucosa induced by chemotherapeutic agents due to their anti-inflammatory and anti-oxidant properties. ^28^ At the same time, *clarified butter* and *cow milk,* in this *enema* preparation, induce a soothing effect due to their *Pitta Shamaka* nature. ^17^

### Strengths and limitations

The combination of all *Ayurveda* medicines and *Panchagavya* therapies induces the *Anulomana* of *Apana Vayu,* which stimulates the *Samana Vayu* and corrects the vitiation of *Kledka Kapha* and *Pachaka Pitta.* ^24^ Therefore, symptoms related to *Amlapitta* and *Sarvanaga Daha (Pitta Prakopa*) were completely subsided. Due to *Kledahara* and *Agnidipaka* properties, it removes the *Rasa Dushti,* resulting in the relief of heart palpitations. As a result, symptoms induced by chemo- and radiotherapy therapy will ease due to the correct combination of *Panchakarma* and *Panchagavya* formulations with their *Vatanulomak, Pittghna, Dahahara, Balya,* and *Rasayana* (immuno-modulators, anti-oxidant, bio-enhancing) properties. Because this is only a single case study, the patient should be treated according to suitable medical guidelines and under the supervision of specialists in the respective domains in the future.

## Conclusion

The present case study shows that the *Ayurvedic* intervention based on *Panchagavya* and *Panchakarma* is effective in subsiding the side-effects induced by the chemo- and radiotherapy through their threefold effects of *Shamana, Bruhana and Mrudu Shodhana.* It is also helpful to increase the compliance rate of the patients towards conventional therapies like chemo-radiotherapy. Further multi-centric clinical trials with large sample sizes are encouraged to establish the significant and comprehensive role of *Panchagavya Chikitsa* with *Panchakarma* in *Ayurveda.* It will become a milestone for *Ayurveda* in oncology for its acceptance at a global level.

### Patient perspective

The patient is satisfied with the treatment protocol and desired outcomes.

### Ethical statement

Written informed consent was received from the patient for publishing this case study and the patient consented to the disclosure of her clinical details. The ethical clearance for the publication of this case study was taken from the local ethical committee at Mahatma Gandhi Ayurveda College, Hospital and Research Centre, Salod (H), Wardha with IRB No. MGAC/IEC/April/2023/102. All procedures and treatments advised and performed in this study were conducted by ethical standards mentioned in the 1964 Declaration of Helsinki, as revised in 2013.

### Glossary

• *Agnidipaka:* increasing appetite
• *Amlapittta Daha:* hyper acidity
• *Annavaha Strotas*: gastro-intestinal channels
• *Anulomana-* correct direction
• *Anuvasana Vasti with Panchgavya Ghrita:* medicated clarified butter enema with Panchagavya
• Apan Vayu: air entity below umbilicus
• *Ashatavidha Parikshana:* eight-fold examination
• *Astha-Majjagata Dushti Vikara:* disease that occurred due to vitiation of toxins at bone and bone marrow
• *Balya:* nutritive
• *Bruhana:* nutritive treatment
• *Chhardi:* vomiting
• *Chikitsa:* treatment
• *Dahahara:* dispelling one’s burning sensation
• *Dhatu:* body tissue
• *Dhatukshayajanaya Vata Vyadhi:* diseases of air entity due to its depletion
• *Dosha:* bio humour
• *Drava Guna* of *Pitta*:liquid nature of fire/hot entity in body
• *Go–Ghrita:* cow clarified butter
• Gomutra: cow urine
• *Granthi* and *Arbuda:* cyst
• *Guda Daha:* burning sensation at the anus
• *Guduch:* Tinospora cordifolia
• *Kamdhenu Gomutra Ark:* distilled cow urine
• *Kantakari:* Solanum xanthocarpum
• *Kapha*: phlegm
• *Kaphaghna*: anti-phlegm
• *Kledahara:* hydrous conetent absorber
• *Kledak Kapha Dushti:* vitiation of phlegm
• *Laghusutshekhar Ras:* herbs-minerals Ayurveda drug combination
• *Matra Vasti:* oil retention enema
• *Mootra Daha:* burning micturition
• *Mrudu Shodhana:* mild detoxification
• *Nimba:* Azadirachta indica
• *Niruha Vasti:* decoction enema
• *Pachak Pitta:* fire/hot entity in abdomen
• *Panchagavya: a* combination of cow milk, curd, clarified butter, urine, and dung
• *Panchagavya Ghrita:* Ghrit prepared from cow milk, curd, clarified butter, urine, and dung
• *Panchakarma:* putative therapy
• *Panchatikta Kshir Vasti:* enema of cow milk treated with *Azadirachta indica* (stem bark), *Tinospora cordifolia* (stem), *Adhatoda vasica* (root), *Solanum xanthocarpum* (root), and *Trichosanthes dioica* (aerial parts)
• *Patol:* Trichosanthes dioica
• *Pitta Dosha:* fire entity or humor in the body
• *Pitta Prakopaka: Pitta dosha* flare-up
• *Pitta Shamaka:* pacification of *Pitta dosha*
• *Pittghna:* anti-bilious
• *Pursihvaha Strotas*: eliminitory channels
• *Ras Dushti*: vitiation of circulatory tissues in the body
• *Rasavaha Strotas*: the channels of the body which convey the first tissue formed from the nutritive juices
• *Rasayana*: immuno-modulators, anti-oxidant, bio-enhancing
• *Ruksha Guna*: dry property
• *Saman Vayu:* air entity at the abdomen
• *Sarvanaga Daha:*burning sensation in all over body
• *Sarvang Daha*: hot flushes
• *Shamana:* pacification treatment
• *Shamana Snehapana:* palliative oleation
• *Shodhana:* purification
• Snigdha Guna: unctuous property
• *Strotas*:circulatory channels
• *Sukshma:* micro
• *Tikshana Shodhana*:extreme putative therapy
• *Ushna Guna:* thermal properties
• *Vamana:* therpeutic emesis
• *Vasa:* Adhatoda vasica
• *Vatanulomak:* the drugs responsible for the change in direction of *Vata Dosha* i.e. *Vata* means to blow or to move like the wind. Containing the elements like air and space,
• *Vatanulomama:* correct the direction of *Vata Dosha*
• *Vatapradhan Pittaj Prakriti:* patient having predominantly air entity followed by fire entity
• *Virechana:*therpeutic purgation
