# Supplementary material for: Case Report: The role of Panchagavya and Panchakarma treatment in the management of radiotherapy and chemotherapy side effects in infiltrative ductal carcinoma
Source: F1000Res. 2024 Apr 8;12:1383. Originally published 2023 Oct 20. [Version 2] doi: 10.12688/f1000research.139233.2 (PMC11544198; doi:10.12688/f1000research.139233.2)
Supplement: Peer review history

# Reviewer report: Reviewer response for version 2

**DOI:** 10.5256/f1000research.164456.r330371

**Published:** 2024-11-07

**Authors:** B Thamarai Selvi

**Affiliations:** 1 Sri Ramakrishna College of Arts and Science for Women, Coimbatore, Tamil Nadu, India

The Panchagavya and Panchakarma therapies outlined here offer a valuable complementary approach in the holistic management of cancer treatment side effects.

 These therapies help mitigate common symptoms like nausea, vomiting, appetite loss, and fatigue associated with chemotherapy and radiotherapy, while also enhancing the immune system and promoting detoxification.

 he reported positive outcomes in the case study suggest that these Ayurvedic therapies can help improve patient quality of life and treatment compliance during cancer therapy. However, further research with larger sample sizes is essential to validate these findings on a wider scale.

Are enough details provided of any physical examination and diagnostic tests, treatment given and outcomes?

Yes

Is the case presented with sufficient detail to be useful for other practitioners?

Partly

Is sufficient discussion included of the importance of the findings and their relevance to future understanding of disease processes, diagnosis or treatment?

Yes

Is the background of the case’s history and progression described in sufficient detail?

Yes

Reviewer Expertise:

Strengths and Limitations: Strengths: Multi-Pronged Action: The combination of various therapies works on multiple aspects of the body, including immunity, digestion, and detoxification. Holistic Approach: Ayurvedic treatments like Panchagavya therapies can serve as complementary treatments that support the body during conventional cancer therapies. Immune Boosting: By enhancing immunity and detoxifying the body, Panchagavya therapies may help improve the patient's overall resilience to treatment and recovery. Limitations: Lack of Large-Scale Evidence: This case study is limited to a single patient, and while the results are promising, larger and more comprehensive studies are necessary to establish the efficacy of these treatments in a broader population. Medical Supervision Required: These therapies should be used under the supervision of trained Ayurvedic practitioners, especially in conjunction with conventional cancer treatments, to avoid any potential adverse effects. Herbal Medicines and ecofriendly dying

I confirm that I have read this submission and believe that I have an appropriate level of expertise to confirm that it is of an acceptable scientific standard.

# Reviewer report: Reviewer response for version 2

**DOI:** 10.5256/f1000research.164456.r330370

**Published:** 2024-10-11

**Authors:** Aishwarya Ashish Joglekar, Sumedh Joshi

**Affiliations:** 1 Samhita Siddhant, All India Institute of Ayurveda, New Delhi, India; 2 Dravyaguna, All India Institute of Ayurveda, New Delhi, India

The authors have successfully provided insights in the treatment of ductal carcinoma of breast. The scientific treatment is done with classical medications and simple therapeutic protocol. 

 Few aspects mentioned below can be incorporated for further understanding of the subject

 1) Incidence and Prevalence of ductal carcinoma in India and/or globally can be expressed in numbers 

 2) Terms like modern science or modern perspective can be avoided and replaced with terms like western biomedicine and conventional medicine

 3) Introduction can be give brief information regarding the Panchagavya treatment

 4) English equivalent for all the sanskrit terms including basti etc should be provided

 5) Pacifier Ghee intake ?? Clarify the statement

 6) The name of GMP company from which the study drugs were used can be mentioned. Authentication certificate can be provided

 7) Term Panchagavya Belief - Not clear

 8) Recent research work done in this field can be mentioned (where in the use of same of drugs for the treatment of ductal carcinoma , ayurvedic treatment of ductal carcinoma can be mentioned)

Are enough details provided of any physical examination and diagnostic tests, treatment given and outcomes?

Partly

Is the case presented with sufficient detail to be useful for other practitioners?

Yes

Is sufficient discussion included of the importance of the findings and their relevance to future understanding of disease processes, diagnosis or treatment?

Yes

Is the background of the case’s history and progression described in sufficient detail?

Partly

Reviewer Expertise:

Ayurveda , Ayurveda Basic principles , endocrinology

We confirm that we have read this submission and believe that we have an appropriate level of expertise to confirm that it is of an acceptable scientific standard, however we have significant reservations, as outlined above.

# Reviewer report: Reviewer response for version 1

**DOI:** 10.5256/f1000research.152493.r220496

**Published:** 2023-12-19

**Authors:** Amit Nakanekar, Payal Rathod

**Affiliations:** 1 Government Ayurved College, Maharashtra, India; 2 Kayachikitsa, Government Ayurved College Nagpur, Nagpur, Maharashtra, India

This Case report nicely describes the importance of Pangavya and Panchkarma treatment in infiltrative ductal carcinoma. I congratulate the authors for successfully treating this challenging case. The authors have nicely explained the case in detail.

 I suggest authors avoid the use of complicated Ayurvedic terminologies to increase the visibility and citation value of the article. Authors need to emphasise the concepts rather than medicines in the discussion section. Authors can compare their current study with previously published articles. Please mention the quality of life scales used for this case report. Provide all the reports as a supplementary material

Are enough details provided of any physical examination and diagnostic tests, treatment given and outcomes?

Yes

Is the case presented with sufficient detail to be useful for other practitioners?

Yes

Is sufficient discussion included of the importance of the findings and their relevance to future understanding of disease processes, diagnosis or treatment?

Partly

Is the background of the case’s history and progression described in sufficient detail?

Yes

Reviewer Expertise:

Ayurveda, Herbal Medicine,

We confirm that we have read this submission and believe that we have an appropriate level of expertise to confirm that it is of an acceptable scientific standard, however we have significant reservations, as outlined above.

# Response

**Published:** 2024-03-30

**Authors:** Punam Sawarkar

Concepts are more emphasized in the discussion section based on reviewer comments and comparisons to prior published papers. A scale for quality of life is employed. WHOQOL-SRPB: scoring and coding for the WHOQOL SRPB field-test instrument: Users Manual, 2012 version, WHO Reference Number: WHO/MSD/MER/Rev.2012.05.

 All the supporting reports are submitted as a supplementary material
